# Report of Adverse Events Associated With Cutaneous Procedures: Update From the CAPER Registry

**DOI:** 10.1002/lsm.70195

**Published:** 2026-09-01

**Authors:** Sabrina S. Smith, Michael D. Yi, Elizabeth Zhang, Joycie Chang, Thanvi Gullapalli, Doroteja Dragovic, Vanessa J. Lazaro‐Camp, Ashish C. Bhatia, Arisa Ortiz, Murad Alam

**Affiliations:** ^1^ Department of Dermatology Feinberg School of Medicine Chicago Illinois USA; ^2^ Oak Dermatology Naperville Illinois USA; ^3^ Department of Dermatology UC San Diego Health San Diego California USA; ^4^ Department of Surgery Northwestern University Feinberg School of Medicine Chicago Illinois USA; ^5^ Department of Otolaryngology Northwestern University Feinberg School of Medicine Chicago Illinois USA; ^6^ Department of Medical Social Sciences Northwestern University Feinberg School of Medicine Chicago Illinois USA

**Keywords:** adverse events, CAPER, cutaneous, procedures, registry, reporting

## Abstract

**Objectives:**

To systematically evaluate submissions to the CAPER database by analyzing procedural context, characteristics of reported adverse events (AEs), and provider involvement in order to identify patterns with potential implications for patient safety.

**Methods:**

A retrospective review of all reports submitted to the CAPER database from March 2021 to August 2025 was conducted, categorizing cases by procedure type, reported AE, and provider involved.

**Results:**

In total, 199 reports describing 298 AEs across 35 various cutaneous procedures were submitted to the CAPER registry from March 2021 to August 2025. Dermatologists performed 62% of reported procedures, while non‐dermatologist physicians, non‐physicians, and unknown providers accounted for 38%. Filler injections, ablative laser resurfacing, and non‐ablative laser treatments were the most frequently reported procedures. Although dermatologists performed most procedures represented in the registry, 51% of reported AEs were associated with procedures performed by non‐dermatology providers. Erythema and scarring were among the most frequently reported AEs, while rare but serious events, including vascular compromise and necrosis, were reported following injectable and energy‐based procedures performed by a variety of provider types, including non‐physicians.

**Conclusions:**

The CAPER registry provides valuable insight into AEs associated with cutaneous procedures and may help identify emerging safety concerns related to new devices or techniques. Broader engagement with AE reporting systems may inform best practices and improve patient safety, particularly as cutaneous procedures increasingly occur outside traditional clinical settings.

## 1 Introduction

Adverse event (AE) registries address procedural safety needs given the limitations of clinical trials and case series, such as detection of rare, delayed, or unexpected AEs. Registries provide post‐market surveillance, signal generation, and hypothesis formation for AEs associated with new or evolving devices or techniques, off‐label use, or combination procedures that may not be captured elsewhere. Registries are of great use in medicine, as rapid innovation, a predominance of outpatient procedures, and operator variability may lead to unintended outcomes. One such registry is the FDA Manufacturer and User Facility Device Experience (MAUDE) database, which has been used to characterize medical device‐related AEs such as those involving cosmetic injectable soft‐tissue fillers and events related to laser and energy‐based devices [1, 2].

The Cutaneous Procedures Adverse Events Reporting (CAPER) registry (https://caper.net/) serves as a nationwide, voluntary database that monitors AEs specific to dermatology [3, 4, 5]. Developed by a steering committee from the American Society for Dermatologic Surgery Association (ASDSA) and Northwestern University, CAPER collects outcomes from surgical, laser, and cosmetic treatments performed by dermatologists, other physicians, and non‐physician clinicians. The CAPER registry was initially only promoted to members of the ASDSA but has since expanded to accept reports of dermatologic AEs from all relevant providers. We sought to systematically evaluate submissions to the CAPER database by examining procedural context, reported AE characteristics, and provider involvement to identify patterns with potential patient safety implications.

## 2 Methods

AE reports submitted to the CAPER database include procedural details (including indication, device or product used, treatment parameters, and who performed the procedure) and AE description (including type, severity, and duration of the AE, timeline from initial detection, response offered for the AE, course of action, and outcome) (Supporting Information S1: Figure 1).

We conducted a retrospective review of all reports submitted to the CAPER database from March 2021 to August 2025. Reports were reviewed and categorized according to the type of procedure performed, the type of reported AE, and the type of provider involved in the procedure.

## 3 Results

From March 2021 to August 2025, 199 reports itemizing 298 AEs pertaining to 35 different types of cutaneous procedures were received (Tables 1 and 2). Of all cases reported, 62% of procedures were performed by dermatologists and 38% by non‐dermatologist physicians, non‐physicians, and unknown providers. Filler injections, ablative laser resurfacing treatments, and non‐ablative laser treatments were the most commonly reported procedures at 18%, 11.5%, and 6%, respectively. Most cases of these three types were performed by dermatologists (Table 1).

**Table 1:** Cutaneous procedures performed by dermatologists versus non‐dermatologists.

Procedure	Cases performed by dermatologists (%)	Cases performed by non‐dermatologists (%)	Total number of cases reported (%)
Filler injection, total	25 (69)	11 (31)	36 (18)
HA filler injection	21 (72)	8 (28)	29 (14.5)
PLLA filler injection	3 (100)	1 (0)	4 (2)
PMMA filler injection	1 (50)	1 (50)	2 (1)
CaHA filler injection	0 (0)	1 (100)	1 (0.5)
Non‐ablative laser treatment	14 (61)	9 (39)	23 (11.5)
Ablative laser resurfacing treatment	8 (67)	4 (33)	12 (6)
Microneedling, unspecified	5 (45)	6 (55)	11 (5.5)
Intense pulsed light	1 (11)	8 (89)	9 (4.5)
Mohs surgery	6 (75)	2 (25)	8 (4)
Radiofrequency microneedling	6 (75)	2 (25)	8 (4)
Botulinum toxin injection	6 (86)	1 (14)	7 (3.5)
Skin flap	4 (67)	2 (33)	6 (3)
Excision	5 (100)	0 (0)	5 (2.5)
Unspecified injection	0 (0)	4 (100)	4 (2)
Thread lift	1 (33)	2 (67)	3 (1.5)
Blepharoplasty	2 (100)	0 (0)	2 (1)
Fat transfer	2 (100)	0 (0)	2 (1)
Ink extraction	0 (0)	2 (100)	2 (1)
Liposuction	1 (50)	1 (50)	2 (1)
Microcoring	1 (50)	1 (50)	2 (1)
Sclerotherapy	2 (100)	0 (0)	2 (1)
Application of topical anesthetic	1 (50)	1 (50)	2 (1)
Benign destruction (i.e., ED&C of SK)	1 (100)	0 (0)	1 (0.5)
Browlift	1 (100)	0 (0)	1 (0.5)
Cryolipolysis	0 (0)	1 (100)	1 (0.5)
Covid booster	0 (0)	1 (100)	1 (0.5)
Dermabrasion	0 (0)	1 (100)	1 (0.5)
Collagenase injection	1 (100)	0 (0)	1 (0.5)
Partial skin graft	1 (100)	0 (0)	1 (0.5)
Intralesional immunotherapy injection	0 (0)	1 (100)	1 (0.5)
Intralesional steroid injection	1 (100)	0 (0)	1 (0.5)
Microfocused ultrasound	1 (100)	0 (0)	1 (0.5)
Microwave energy	0 (0)	1 (100)	1 (0.5)
Non‐invasive muscle stimulation	0 (0)	1 (100)	1 (0.5)
Neck lift	1 (100)	0 (0)	1 (0.5)
Phenol peel	0 (0)	1 (100)	1 (0.5)
Plasma resurfacing	0 (0)	1 (100)	1 (0.5)
Superficial radiation therapy	1 (100)	0 (0)	1 (0.5)
Totals	123 (62)	75 (38)	198 (100)

Abbreviations: ED&C, electrodesiccation and curettage; IPL, intense pulsed light; SK, seborrheic keratosis.

**Table 2:** Adverse events reported by dermatologists versus non‐dermatologists.

Adverse event	Number of AEs performed by dermatologists (%)	Number of AEs performed by non‐dermatologists (%)	Total number of AEs reported (%)
Erythema	13 (37)	22 (63)	35 (12)
Scar	6 (23)	20 (77)	26 (9)
Nodule	13 (59)	9 (41)	22 (7)
Swelling	11 (50)	11 (50)	22 (7)
Hyperpigmentation	7 (35)	13 (65)	20 (7)
Blistering	7 (39)	11 (61)	18 (6)
Pain	10 (63)	6 (37)	16 (5)
Skin texture alteration	3 (21)	11 (79)	14 (5)
Induration	8 (62)	5 (38)	13 (4)
Infection	8 (62)	5 (38)	13 (4)
Vascular compromise	7 (63)	4 (37)	11 (4)
Contour irregularity	2 (20)	8 (80)	10 (3)
Hypopigmentation	4 (40)	6 (60)	10 (3)
Necrosis	6 (60)	4 (40)	10 (3)
Burn	1 (25)	3 (75)	4 (1)
Ulceration	3 (100)	0 (0)	3 (1)
Abrasion	0 (0)	2 (100)	2 (0.7)
Discoloration	0 (0)	2 (100)	2 (0.7)
Granuloma	2 (100)	0 (0)	2 (0.7)
Hematoma	2 (100)	0 (0)	2 (0.7)
Hypersensitivity	1 (50)	1 (50)	2 (0.7)
Inflammation	0 (0)	2 (100)	2 (0.7)
Keloid	2 (100)	0 (0)	2 (0.7)
Transient facial paralysis	2 (100)	0 (0)	2 (0.7)
Abscess	1 (100)	0 (0)	1 (0.3)
Acne	1 (100)	0 (0)	1 (0.3)
Bullae	0 (0)	1 (100)	1 (0.3)
Crusted erosions	1 (100)	0 (0)	1 (0.3)
Fat atrophy	1 (100)	0 (0)	1 (0.3)
Festooning	0 (0)	1 (100)	1 (0.3)
Needle retraction failure	1 (100)	0 (0)	1 (0.3)
Nerve injury	1 (100)	0 (0)	1 (0.3)
Partial graft failure	1 (100)	0 (0)	1 (0.3)
Pustule	1 (100)	0 (0)	1 (0.3)
Rash	0 (0)	1 (100)	1 (0.3)
Seroma	1 (100)	0 (0)	1 (0.3)
Short duration of action	1 (100)	0 (0)	1 (0.3)
Sialocele	1 (100)	0 (0)	1 (0.3)
Sloughing	1 (100)	0 (0)	1 (0.3)
Soreness	0 (0)	1 (100)	1 (0.3)
Urticaria	1 (100)	0 (0)	1 (0.3)
Wound dehiscence	1 (100)	0 (0)	1 (0.3)
Other, total	8 (86)	2 (14)	10 (3)
Other: Brow ptosis	2 (100)	0 (0)	2 (0.7)
Other: Purpura	2 (100)	0 (0)	2 (0.7)
Other: Migraine	2 (100)	0 (0)	2 (0.7)
Other: Chemical eye injury	1 (100)	0 (0)	1 (0.3)
Other: Corneal inflammation	0 (0)	1 (100)	1 (0.3)
Other: Wrong site surgery	1 (100)	0 (0)	1 (0.3)
Other: Lip ptosis	0 (0)	1 (100)	1 (0.3)
Total	146 (49)	152 (51)	298 (100)

Although dermatologists performed most procedures reported in the CAPER registry, a substantial number of reports were associated with procedures performed by non‐dermatologists (Table 2). The most frequently reported AEs were erythema and scarring. Of the reported cases of erythema and scarring in the registry, 63% and 77%, respectively, were associated with procedures performed by non‐dermatology providers (Table 2). The most concerning AEs reported included vascular compromise and necrosis, although the frequency of these was much lower. Cases that resulted in vascular compromise included calcium hydroxyapatite and hyaluronic acid filler injections of the cheek, a non‐FDA approved pharmaceutical injection containing hyaluronic acid, perioral and lip hyaluronic acid filler injections, nasolabial fold hyaluronic acid filler injections, and temple hyaluronic acid filler injections. In all of these cases, the vascular compromise resolved spontaneously or with hyaluronidase treatment. Cases that resulted in necrosis included partial graft failure on the scalp with necrotic skin, necrosis of the fascia and muscle of the forearm following skin cancer excision, slight sloughing of the mucosal lip after hyaluronic acid filler injection, open ulcerations with frank impetiginization following plasma resurfacing, ulceration of the shin and calf following sclerotherapy for leg veins, complete graft failure and ulceration on the calf following Mohs surgery, and severe burns and skin sloughing following intense pulsed light (IPL) treatment. These cases resolved with treatments such as skin substitutes, biologic dressings, antibiotics, or hyaluronidase administration.

Certain procedures, including “unspecified injections,” ink extractions, and IPL, were more commonly associated with concerning AEs. The injections involved unknown fillers, an “antiox facial solution” intended only for topical use, and a polynucleotide (PN) injectable derived from marine DNA marketed for periorbital rejuvenation. The unspecified injections were administered by either unidentified providers or non‐physicians in med‐spas and associated with erythema, nodules, and induration. The ink extraction procedures were performed by tattoo artists using a non‐laser removal method (Tatt2Away) and were associated with permanent scarring (Supporting Information S2: Table 1). Cases associated with light treatment, for example, IPL, were more frequently performed by non‐physicians, which was not unexpected as IPL treatments are typically performed in med‐spas, and improper treatment often causes striping, burning, or blistering [6, 7].

## 4 Discussion

Given the very large number of cutaneous procedures performed per year in the United States, the modest number of reports in the CAPER database reflects the number of events submitted to this voluntary reporting system and does not allow estimation of the true incidence of AEs. Notwithstanding the voluntary nature of the reporting system, the presented data also highlight several concerning events which appear to arise in nontraditional clinical environments, such as med‐spas or settings involving non‐physician providers. While most complications were mild and self‐remitting, reports of serious AEs such as vascular compromise and necrosis are particularly concerning and may represent sentinel events that underestimate their true prevalence.

Registries such as CAPER are useful tools for tracking events outside of the expected clinical course, including rare or unusual complications and delayed AEs. Red‐flag events may result in severe, progressive, or prolonged adverse outcomes; functional or cosmetic morbidity; or infectious, vascular, neurologic, or systemic involvement. Timely submission after event recognition is imperative so that safety concerns are recognized and addressed promptly. Ongoing surveillance through structured reporting systems is therefore essential to identify emerging safety patterns and inform targeted quality improvement efforts.

One limitation of the CAPER database is the disproportionately high reporting from dermatologists, who may be more aware of the registry given that it has been co‐managed by and was originally promoted to the ASDSA, a professional society representing dermatologic surgeons. Although the registry has since expanded to accept dermatologic AE reports from all relevant providers, this may still introduce reporting bias. AEs associated with other provider groups are likely underreported in the CAPER registry [4, 5]. Additionally, the registry does not provide denominator data for the total number of procedures performed by each provider type, making comparisons of complication frequency unreliable.

Broader awareness of the CAPER registry may help mitigate this bias and yield more complete data that would help further improve patient safety. Registry reporting should be emphasized as non‐punitive, but rather quality improvement focused, to maximize reporting practices across provider groups. Future efforts may focus on integration into routine clinical and institutional workflows to encourage broad participation and data completeness.

## 5 Conclusion

The CAPER registry is useful as reports of AEs associated with new procedures or devices may lead to prompt attention by physicians, manufacturers, and regulators to address underlying safety concerns. Reports to the CAPER registry reveal that AEs following cutaneous procedures remain an important patient safety issue, particularly for procedures performed in nontraditional clinical settings. Finally, broader engagement with AE registries like CAPER may guide best practices and enhance patient safety as cutaneous procedures continue to evolve.

## Author Contributions

Study concept and design: M.A. Acquisition, analysis, and interpretation of data: S.S.S., M.D.Y., E.Z., T.G., J.C., D.D., V.J.L., K.H., and M.A. Drafting of the manuscript: S.S.S. and M.D.Y. Administrative, technical, or material support: K.H. Statistical analysis: S.S.S., M.D.Y., and M.A. Critical revision of the manuscript for important intellectual content: E.Z., T.G., J.C., D.D., V.J.L., K.H., and M.A. Study supervision: M.A. All authors had access to the information to certify its accuracy.

## Disclosure

IRB approval status: Not applicable. Clinicaltrials.gov (or equivalent) listing (if applicable): Not applicable. Reprint requests: Murad Alam, MD, MBA, MSCI.

## Consent

The authors have nothing to report.

## Conflicts of Interest

All financial interests (including pharmaceutical and device product[s]):

1. Employment: Dr. Alam is employed at Northwestern University.
2. Consultancies: None.
3. Honoraria: None.
4. Speaker's bureau: None.
5. Stock ownership or options: None.
6. Expert testimony: None.
7. Grants: Northwestern University has a clinical trials unit that receives grants from various corporate and governmental entities to perform clinical research. In all cases, grants have been provided to Northwestern University and not to Dr. Alam directly, and Dr. Alam has not received any salary support from these grants to the University.
8. Patents filed, received, pending, or in preparation: None.
9. Royalties: None.
10. Donation of medical equipment: None.

## Use of AI

AI was not used.

## Supporting information


Supporting File 1

Supporting File 2

## Data Availability

The data that support the findings of this study are available on request from the corresponding author. The data are not publicly available due to privacy or ethical restrictions.
