# Supplementary material for: Report of Adverse Events Associated With Cutaneous Procedures: Update From the CAPER Registry
Source: Lasers Surg Med. 2026 Sep 1;58(9):796–801. doi: 10.1002/lsm.70195 (PMC13649085; doi:10.1002/lsm.70195)
Supplement: Supplementary file 1 [file LSM-58-796-s001.docx]

**Supplemental Figure 1.** How to report adverse events to the CAPER registry*

Step 1: Identify Reportable Event - Any adverse event following a cutaneous procedure - Includes expected, unexpected, mild, or severe events

Step 2: Access Reporting Platform - CAPER registry adverse event form: https://caperphys.formstack.com/forms/caper_physician

Step 3: Enter Adverse Event Information - Includes type of AE, severity of AE, duration of AE, and outcome - Document drug or product information, where applicable

Step 4: Enter Basic Patient Information - Includes demographics (de-identified) and relevant medical history

Step 5: Enter Provider Information - Includes title, original procedure setting location, and AE treatment or correction details

Step 6: Enter Reporter Information - Includes your details and attestation

Step 7: Submit Report

**Not all fields are mandatory. Required fields are indicated by an asterisk (*), and the CAPER form will automatically prompt completion of any missing required fields before advancing to the next section.*
