# Supplementary material for: Report of Adverse Events Associated With Cutaneous Procedures: Update From the CAPER Registry
Source: Lasers Surg Med. 2026 Sep 1;58(9):796–801. doi: 10.1002/lsm.70195 (PMC13649085; doi:10.1002/lsm.70195)
Supplement: Supplementary file 2 [file LSM-58-796-s002.docx]

**Supplemental Table 1.** Adverse Events of Procedures Commonly Performed by Non-Physicians

| **Procedure** | **Cases Performed by Physicians (%)** | **Cases Performed by Non-Physicians (%)** | **Total Number of Cases Reported (%)** | **Most Common Adverse Events Reported per Procedure, n (%)** |
| --- | --- | --- | --- | --- |
| Unspecified Injection | 0 (0) | 4 (100) | 4 (100) | Erythema, 2 (18) Nodule, 2 (18) Induration, 2 (18) Swelling, 2 (18) Scar, 2 (18) |
| Ink Extraction | 0 (0) | 2 (100) | 2 (100) | Hyperpigmentation, 2 (40) Scar, 2 (40) Erythema, 1 (20) |
| Intense Pulsed Light | 1 (11) | 8 (89) | 9 (100) | Blistering, 3 (18) Burn, 3 (18) Erythema, 2 (12) Swelling, 2 (12) Hyperpigmentation, 2 (12) |
| Thread Lift | 1 (33) | 2 (67) | 3 (100) | Pain, 2 (25) Erythema, 1 (12.5) Scar, 1 (12.5) Hyperpigmentation, 1 (12.5) Infection, 1 (12.5) |
| Microneedling, Unspecified | 5 (45) | 6 (55) | 11 (100) | Erythema, 3 (27) Swelling, 2 (18) Hyperpigmentation, 2 (18) Skin texture alteration, 2 (18) Pain, 1 (9) |
| Non-Ablative Laser Treatment | 14 (61) | 9 (39) | 23 (100) | Blistering, 10 (24) Hyperpigmentation, 8 (20) Hypopigmentation, 5 (12) Pain, 4 (10) Scar, 3 (7) |
| Ablative Laser Resurfacing Treatment | 8 (67) | 4 (33) | 12 (100) | Erythema, 6 (22) Scar, 4 (15) Hyperpigmentation, 3 (11) Infection, 3 (11) Contour irregularity, 3 (11) |
| Filler Injection, Total | 25 (69) | 11 (31) | 36 (100) | Nodule, 13 (20) Vascular compromise, 9 (14) Erythema, 7 (11) Swelling, 7 (11) Induration, 5 (8) |
| HA Filler Injection | 21 (72) | 8 (28) | 29 (100) | Nodule, 9 (17) Vascular compromise, 9 (17) Erythema, 7 (13) Swelling, 7 (13) Scar, 3 (6) |
| PLLA Filler Injection | 3 (100) | 0 (0) | 3 (100) | Nodule, 2 (29)  Induration, 2 (29) Skin texture alteration, 1 (14) Granuloma, 1 (14)  Contour irregularity, 1 (14) |
| PMMA Filler Injection | 1 (50) | 1 (50) | 2 (100) | Nodule, 2 (50) Inflammation, 1 (25) Soreness, 1 (25) |
| CaHA Filler Injection | 0 (0) | 1 (100) | 1 (100) | Vascular compromise, 1 (50) Lip ptosis, 1 (50) |
| Radiofrequency Microneedling | 5 (71) | 2 (29) | 7 (100) | Erythema, 2 (17) Scar, 1 (8) Nodule, 1 (8) Hyperpigmentation, 1 (8) Pain, 1 (8) |
